# Rhamnolipids and fengycins, very promising amphiphilic antifungal compounds from bacteria secretomes, act on *Sclerotiniaceae* fungi through different mechanisms

**DOI:** 10.3389/fmicb.2022.977633

**Published:** 2022-09-29

**Authors:** Camille Botcazon, Thomas Bergia, Didier Lecouturier, Chloé Dupuis, Alice Rochex, Sébastien Acket, Philippe Nicot, Valérie Leclère, Catherine Sarazin, Sonia Rippa

**Affiliations:** ^1^Unité de Génie Enzymatique et Cellulaire, CNRS UMR 7025, Sorbonne Universités, Université de Technologie de Compiègne, Compiègne, France; ^2^Charles Viollette Institute, UMRt BioEcoAgro 1158-INRAe, Métabolites Secondaires d’Origine Microbienne, Université de Lille, Université de Liège, Lille, France; ^3^Centre de Recherche PACA, Domaine Saint Maurice, Unité de Pathologie Végétale, INRAe, Avignon, France; ^4^Unité de Génie Enzymatique et Cellulaire, CNRS UMR 7025, Université de Picardie Jules Verne, Amiens, France

**Keywords:** rhamnolipids, fengycins, *Sclerotinia sclerotiorum*, *Botrytis cinerea*, ergosterol, antifungal lipids

## Abstract

Rhamnolipids (RLs) and fengycins (FGs) are amphiphilic lipid compounds from bacteria secretomes proposed to replace synthetic pesticides for crop protection. They both display plant defense triggering properties and direct antimicrobial activities. In particular, they have well reported antifungal effects against phytopathogenic fungi. RLs and FGs are considered to act through a direct interaction with membrane lipids and a destabilization of microorganism plasma membrane, thereby limiting the risk of resistance emergence. The main objective of this work was to gain insights in the antimycelial mode of action of these metabolites to promote them as environment and human health friendly biocontrol solutions. Their biocidal effects were studied on two *Sclerotiniaceae* fungi responsible for diseases in numerous plant species worldwide. We show here that different strains of *Botrytis cinerea* and *Sclerotinia sclerotiorum* have opposite sensitivities to RLs and FGs on plate experiments. Overall, *B. cinerea* is more sensitive to FGs while *S. sclerotiorum* is more sensitive to RLs. Electron microscopy observations demonstrated that RLs induce mycelial destructuring by asperities emergence and hyphal fusions whereas FGs promote swelling and formation of vesicle-like structures due to vacuole fusions and autophagy. Permeability studies, phosphatidylserine externalization and reactive oxygen species production assessments showed a programmed cell death triggering by RLs at medium concentrations (until 50 μg mL^−1^) and necrosis characteristics at higher concentration. Programmed cell death was always observed on hyphae treated with FGs. Quantifications of mycelial ergosterol content indicated that a higher ergosterol rate in *S. sclerotiorum* correlates with increasing sensitivity to RLs. Oppositely, a lower ergosterol rate in *B. cinerea* correlates with increasing sensitivity to FGs, which was confirmed by ergosterol biosynthesis inhibition with tebuconazole. This gain of knowledge will help to better understand the mode of action of RLs and FGs to fight specific plant fungal diseases.

## Introduction

Plant pathogens are responsible for important yield losses in many crops, ranging from 10 to 40% (Savary et al., 2019). *Botrytis cinerea* and *Sclerotinia sclerotiorum* are two closely related plant pathogens belonging to the *Sclerotiniaceae* family. They are, respectively, causing the grey mold and the white mold diseases in more than 400 plant species including soybean, canola and sunflower at various developmental stages (Lu, 2003; Williamson et al., 2007). Chemical pesticides are widely used to fight these pests and assure yields. They directly act on essential fungal functions such as respiration, sterol biosynthesis or cell division by targeting proteins which led to the emergence of pathogen resistances by gene mutations (Leroux, 2003). Furthermore, chemical pesticides have potential detrimental effects on human health and environment (Lu, 2003; Williamson et al., 2007). The search for alternative solutions is currently necessary to reduce or replace the chemical fungicides used to protect crops from these fungi, in order to propose more sustainable productions.

Some natural amphiphilic compounds from bacterial secretomes are very promising for crop protection. Glycolipid rhamnolipids (RLs) and lipopeptide fengycins (FGs), produced mainly by *Pseudomonas* and *Bacillus* bacteria species respectively, have very interesting properties to protect plants. They both display plant defense stimulation activities and have direct antimicrobial effects. In particular, they both have antifungal properties against phytopathogenic fungi (Crouzet et al., 2020). RLs are composed of one (mono-rhamnolipids) or two (di-rhamnolipids) rhamnose glycosyl polar heads linked through a *β*-glycosidic bond to one or two 3-hydroxyfatty acid hydrophobic tails. FGs are composed of a *β*-hydroxy fatty acid linked to a peptide part comprising 10 amino acids, 8 of which being organized in a cyclic structure. RLs are non-toxic for plants (Monnier et al., 2018) and are weakly or non-toxic to mammalian cells (Falqueto et al., 2021). These molecules display a low ecotoxicological potential (Johann et al., 2016). FGs, as lipopeptides, are not associated with any phytotoxicity or adverse effect on the integrity of plant cells (Ongena and Jacques, 2008). Both RLs and FGs are biodegradable (Mohan et al., 2006; Crouzet et al., 2020). As membranotropic compounds, their mode of action is to induce a loss of membrane integrity in microorganisms (Crouzet et al., 2020). This is less likely to induce the development of pathogen resistance compared to antifungal compounds affecting the synthesis of specific membrane components through mutations on target sites of action (Avis, 2007).

It has already been shown that RLs inhibit mycelial growth of *B. cinerea* but have no effect on spore germination (Kim et al., 2000; Abalos et al., 2001; Haba et al., 2003; Varnier et al., 2009; Sha et al., 2012; Monnier et al., 2018; Sanchez et al., 2019). Morphology alterations have been also described on *Fusarium oxysporum* treated with RLs (Borah et al., 2015). Structural modifications and alterations of membrane permeability have been reported on *Alternaria alternata* (Yan et al., 2015). FGs display antifungal activities against *B. cinerea* and *S. sclerotiorum* by inhibiting mycelial growth (Vanittanakom et al., 1986; Alvarez et al., 2012; Zhao et al., 2013; Farzand et al., 2019). FGs also induce morphological changes in the mycelium, such as deformities and swelling (Vanittanakom et al., 1986; Alvarez et al., 2012; Farzand et al., 2019). Swelling and morphological changes induced by FGs are also reported on other mycelial fungi such as *Schizophillium commune*, *Magnaporthe grisea* and *Venturia inaequalis* (Tang et al., 2014; Zhang and Sun, 2018; Desmyttere et al., 2019). FGs induce cell death on *S. commune* and *M. grisea* (Tang et al., 2014; Zhang and Sun, 2018).

The mode of action of RLs is proposed to result from interactions with the plasma membrane of target cells and a detergent effect (Stanghellini and Miller, 1997). The lipid composition of biomimetic plasma membrane modulates their effect on membrane dynamic (Aranda et al., 2007; Sur et al., 2018). In particular, the fungal sterol ergosterol, is likely to play a role in the RL impact on membrane models by increasing their fluidity (Monnier et al., 2019). FGs likely act by making the plasma membrane of the target cell more permeable. They have been proposed to act in a detergent-like manner *via* solubilization of the lipid bilayer and formation of transmembrane pores that lead to permeability changes, depending on the concentration (Deleu et al., 2008; Zakharova et al., 2019). Differences of sensitivities of different fungal pathogens to FGs could also be related to their ergosterol content (Wise et al., 2014). Nevertheless, in the case of FGs, membrane models seem to be stabilized by ergosterol when exposed to FGs in biophysical studies (Mantil et al., 2019). These studies tend to show an opposite effect of ergosterol on the activities of RLs and FGs on fungal membranes.

To our knowledge, no studies were conducted to investigate and compare the effects of RLs and FGs on the same fungi despite the similarities of their mode of action targeting the plasma membrane. Regarding the antimycelial and cellular effects, it is difficult to compare RLs and FGs in the former different studies because they were performed with metabolite mixtures of different compositions, on different fungi, with different strains and in different growth conditions. Furthermore, the opposite effect of the ergosterol content has been mainly reported on membrane models in biophysical studies and need to be confirmed *in vivo* with both lipid-based amphiphilic compounds.

In this work, we studied and compared the antifungal effects of RLs and FGs on different strains of *B. cinerea* and *S. sclerotiorum* grown in the same conditions. We analysed their activities at different concentrations on hyphal cells by microscopy observations. We also studied the ergosterol content of the fungi to unravel the mode of action of these two very promising membranotropic antifungal compounds.

## Materials and methods

### FGs and RLs purification and preparation

Fengycins (synonym plipastatins) were produced by fermentation of *Bacillus subtilis* Bs2504 which is a fengycin producer derivative of *B. subtilis* 168 (Ongena et al., 2007). Bs2504 was grown in modified Landy medium (20 g L^−1^ glucose, 5 g L^−1^ glutamic acid, 1 g L^−1^ yeast extract, 1 g L^−1^ K_2_HPO_4_, 0.5 g L^−1^ MgSO_4_.7H_2_O, 0.5 g L^−1^ KCl, 1.6 mg L^−1^ CuSO_4_.5H_2_O, 1.2 mg L^−1^ MnSO_4_.H_2_O, 0.4 mg L^−1^ FeSO_4_.7H_2_O, 100 mM 3-morpholinopropane-1-sulfonic acid (MOPS) buffer, pH 7.0) at 30°C under 160 rpm shaking. The strain was taken from a − 80°C frozen stock and transferred onto solid Tryptic Soy Agar medium. A single colony was used to inoculate a 500 ml flask containing 100 ml of medium and then incubated for 24 h. This preculture was then used to inoculate the cultures which were carried out in 5 l flasks containing 1 l of medium for 72 h. FGs were separated and quantified by HPLC as described in Vassaux et al. (2021). For FGs composition analyses, the HPLC system was coupled to a mass spectrometer detector Synapt G2-Si-HESI-Quadripol-ToF (Waters, Massachussetts, United States) piloted by the MassLynx 4.1 software (Waters). Analyses showed that FGs obtained were pure at 97% and were composed of fengycins A and B with saturated or unsaturated fatty acid chain from C15 to C18 carbons. From this composition characterization, a molar weight of 1488.5 g mol^−1^ was estimated. RLs used in this study were a semipurified rhamnolipid mixture produced by *Pseudomonas aeruginosa* fermentations (RL90-A, AGAE Technologies, Oregon, United States). The 85% purity of the product was previously analysed (Monnier et al., 2020). The mix contains 66% of mono-rhamnolipids and 34% of di-rhamnolipids with a majority of C10 fatty acid chains. A molar weight of 534 g mol^−1^ was estimated from this composition characterization.

Stock solutions of RLs and FGs were prepared at a concentration of 10 g L^−1^ in distilled water. Solutions were then diluted directly in fungal growth medium to reach the expected concentrations. To compare the effects of the two compounds in around equimolar concentrations based on the estimations of molar weight and purity, RL doses were fixed at twice the doses of FGs.

### *Sclerotinia sclerotiorum* and *Botrytis cinerea* strains and culture conditions

The *S. sclerotiorum* strains Ss25 and Ssc51 were kindly provided by the technical institut Terres Inovia (Grignon, France). The *S. sclerotiorum* strain Ss123 and the *B. cinerea* strains Bc1, Bc26, Bc82 were provided by the French National Institute for Agriculture, Food and Environment (INRAe, Montfavet, France). Twenty-five μL of a *B. cinerea* spore suspension (stored at −20°C at 1.3 10^6^ ml^−1^) were first germinated on Potato Dextrose Agar 39 g L^−1^ plates (PDA, VWR Chemicals, France). *S. sclerotiorum* sclerotia were cut in half and the internal face of one piece was placed on 39 g L^−1^ PDA plates. The pre-cultures were incubated 4 days at 19°C in darkness. Mycelium agar plugs were then excised with a 6 mm diameter cork borer, transferred on fresh PDA plates and incubated for 2 days at 19°C in darkness, until the colonies occupied half of the plate surface. For microscopy observations, mycelium was scraped off the plates with an inoculating loop and inoculated in 2 ml of 12 g L^−1^ Potato Dextrose Broth (PDB, Sigma-Aldrich, Missouri, United States) in 12-well plates and incubated 24 h at 25°C in darkness, with 60 rpm shaking. To quantify ergosterol content, 5 ml of PDB were inoculated by the scrapping method and incubated in the same conditions for 4 days.

### Antifungal activities assessment

Antifungal activities were determined by measurements of mycelium radial growth on solid medium plates. Six mm diameter plugs were excised from 2-day-old PDA cultures of *B. cinerea* or *S. sclerotiorum*. The plugs were transferred to Petri dishes containing PDA supplemented or not with RLs and FGs at expected concentrations. Forty-eight hours post-inoculation, 2 perpendicular diameters of the mycelial growth were measured on 3 plates per condition. The percentage of growth inhibition (I) was computed according to the formula:


$$I=\frac{100\;\left(Gr_{c}-Gr_{\mathrm{AC}}\right)}{Gr_{c}}$$

Gr_C_ and Gr_AC_ were the average fungal colony diameter on the control plates and on the plates with an added compound, respectively. The presented data correspond to values from 3 biological independent replicates per strain.

Sensitivity to tebuconazole (TN, Sigma-Aldrich), which inhibits the lanosterol-14-alpha-demethylase involved in the synthesis of 4,4-dimethyl cholesta-8,12,24-trienol, a precursor of ergosterol (Price et al., 2015), was evaluated in same conditions.

### Microscopy observations

Fungal mycelium cultivated in 12-well plates was treated or not by RLs or FGs during 24 h in PDB. Scanning electron microscopy was performed with a FEI Quanta-250 scanning electron microscope. Mycelium was thoroughly washed with distilled water, observed on a Peltier module at environmental pressure (450 to 550 Pa) and the images were magnified 1,000x.

To stain mycelium for confocal microscopy, 1 ml of growth medium was first removed from each well before staining to reduce the volumes of treatment. For permeability assessment, mycelium was washed and stained 15 min with 1 μM SYTOX green (Molecular Probes, Invitrogen, California, United States) in 1 ml of PDB. To determine cell death, mycelium was stained 15 min with 5 μl Alexa Fluor® 488 annexin V (Molecular Probes, Invitrogen) and 1 μl of 100 μg mL^−1^ propidium iodide (Molecular Probes, Invitrogen). The process was stopped by adding 400 μl of annexin buffer (Molecular Probes, Invitrogen) in each well. To assess lipid droplets, mycelium was stained 10 min with 10 μg mL^−1^ nile red (Molecular Probes, Invitrogen) at room temperature and washed twice in PBS as described in Chang et al. (2015). To observe the production of reactive oxygen species (ROS), mycelium was stained 45 min with 1 mM dihydroethidium (DHE, Molecular Probes, Invitrogen). All observations were performed with a LSM-710 Zeiss confocal microscope and Zen Black 2.1 SP1 software (Zeiss, Germany). Excitation wavelengths for the different fluorochromes were 488 nm for SYTOX green, Alexa Fluor®, annexin V, propidium iodide, nile red and 518 nm for DHE. Emission wavelengths were 523 nm, 530 nm, 575 nm, 636 nm and 606 nm, respectively. All the confocal microscopy images were magnified 630x.

For permeability evaluation by Evans blue staining, mycelium was washed with phosphate-buffered saline (PBS, 137 mM sodium chloride, 2.7 mM potassium chloride, 10 mM sodium phosphate dibasic, 1.8 mM potassium phosphate monobasic), stained with Evans blue (0.025% in PBS) 10 min, then washed 3 times by gentle manual shaking in 40 ml of PBS. Quantifications were performed with a numerical microscope VHX-6000 (Keyence, Japan). Automatic area measurement option was used and the ratio (%) between no stained areas and stained areas was defined on at least 10 pictures for one treatment and repeated at least twice. Observed samples were magnified by 500.

### Ergosterol extractions and quantification By GC–MS

All extraction steps were performed with samples protected from light, to avoid any ergosterol degradation. After a 2-day lyophilisation of the mycelium, ergosterol was extracted and analysed following the method of Debieu et al. (2013). Each sample contained 5 batches of mycelium cultures grown in 5 ml of PDB. Samples were grounded with mortar and pestle in liquid nitrogen and saponified for 2 h at 70°C in 6% KOH in methanol, to release the esterified ergosterol. Each sample was then extracted 3 times in H_2_O:hexane (1,6, v,v). The extractions were dried and acetylated by adding 100 μl of 1 M acetic anhydride and 50 μl of 1 M pyridine. The reaction took place overnight in darkness. The samples were evaporated and re-suspended in 1 M heptane for gas chromatography analyses coupled with ultra-high resolution mass spectrometry (GC-UHRMS). GC-UHRMS analysis was done using a Thermo GC Orbitrap (Q-ExactiveTM, Thermo Fisher, Massachussetts, United States) equipped with a SGE Analytical Science BP-5MS G1902 (30 m x 0.25 mm, 0.20 μm, Pennsylvania, United States). One μl was injected into the GC. The injection temperature was set at 250°C, and the injection mode was set to split with a ratio of 1:10. Helium was used as the carrier gas at 1.10 ml min^−1^. The GC oven temperature was initially held at 80°C for 2 min and then was ramped at 5°C per min to 300°C. After 2 min at 300°C, the column was ramped at 10°C per min to 350°C and held for 2 min. Transfer line was heated to 250°C and the mass spectrometer source temperature was maintained at 250°C. Ionization was carried out in electronic impact at 70 eV. Data were acquired in full scan mode with a scan range from 50 to 500 m/z in ultra-high resolution (60,000). XCalibur software (Thermo Fischer) allowed to control the parameters of the machine and to perform the data processing. Peaks were annotated using 2014 NIST mass spectral library and an in house UHRMS database. This database was made from standards purchased commercially (ergosterol, cholesterol) injected into the GC-UHRMS. For analyses, an internal standard (cholesterol, Sigma-Aldrich) was acetylated with the same method as the mycelium samples and used to quantify the ergosterol contents, by comparison of ratio between peak of ergosterol/peak of internal standard for each replicate and strain of fungi. Four to six replicates were analysed for each strain of each fungus.

### Statistical analyses

Statistical differences between the growth inhibitions, in ergosterol contents as well as the quantification of stained cells areas by Evans blue assessment were analysed by pairwise t test (*p* < 0.05), with value of p adjustment using the Bonferoni method. Distinct groups were designated by different letters. Statistical analyses were performed using R-software (R core Team, 2014).

## Results

### Effect of RLs and FGs on mycelial growth of *Botrytis cinerea* and *Sclerotinia sclerotiorum*

**Figure 1:**
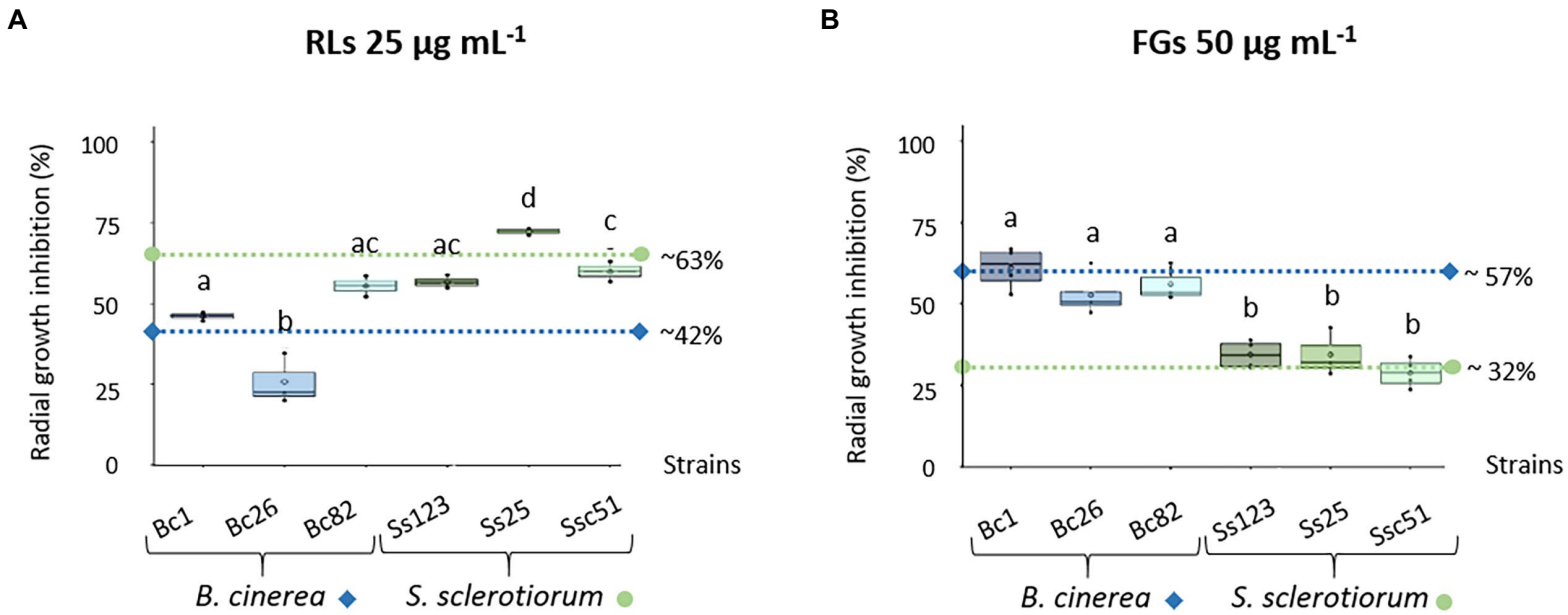
Sensitivity of different *Botrytis cinerea* (Bc1, Bc26, Bc82) and *Sclerotinia sclerotiorum* (Ss123, Ss25, Ssc51) strains to RLs and FGs. Radial growth inhibition of different *B. cinerea* and *S. sclerotiorum* strains in response to 25 μg mL^−1^ RLs **(A)** or 50 μg mL^−1^ FGs **(B)**. Data correspond to means of three biological repetitions, whiskers correspond to inter-quartile ranges and letters indicate significant differences (*p* < 0.05) between the groups. Dotted lines correspond to the mean inhibition growth of each fungus species, diamonds and circles for *B. cinerea* and *S. sclerotiorum*, respectively.

To assess the sensitivity of *B. cinerea* and *S. sclerotiorum* to RLs and FGs, 3 strains of each fungal species were grown on PDA plates to perform radial growth inhibition experiments. RLs and FGs were applied at 25 μg mL^−1^ and 50 μg mL^−1^ respectively, overall corresponding to equimolar concentrations of both compounds considering estimations of purity and metabolite compositions. RLs had a mostly greater effect on *S. sclerotiorum* strains with a 63% average of radial growth inhibition. The average effect of RLs on *B. cinerea* strains was lower with an inhibition of about 42% (Figure 1A). On the contrary, FGs had a greater effect on *B. cinerea* strains with about 57% inhibition and only 32% inhibition on the *S. sclerotiorum* strains. The effect of FGs appeared less scattered among the 3 strains of each fungus species, without any significant difference (Figure 1B).

### Structural alterations of hyphae induced by RLs and FGs

**Figure 2:**
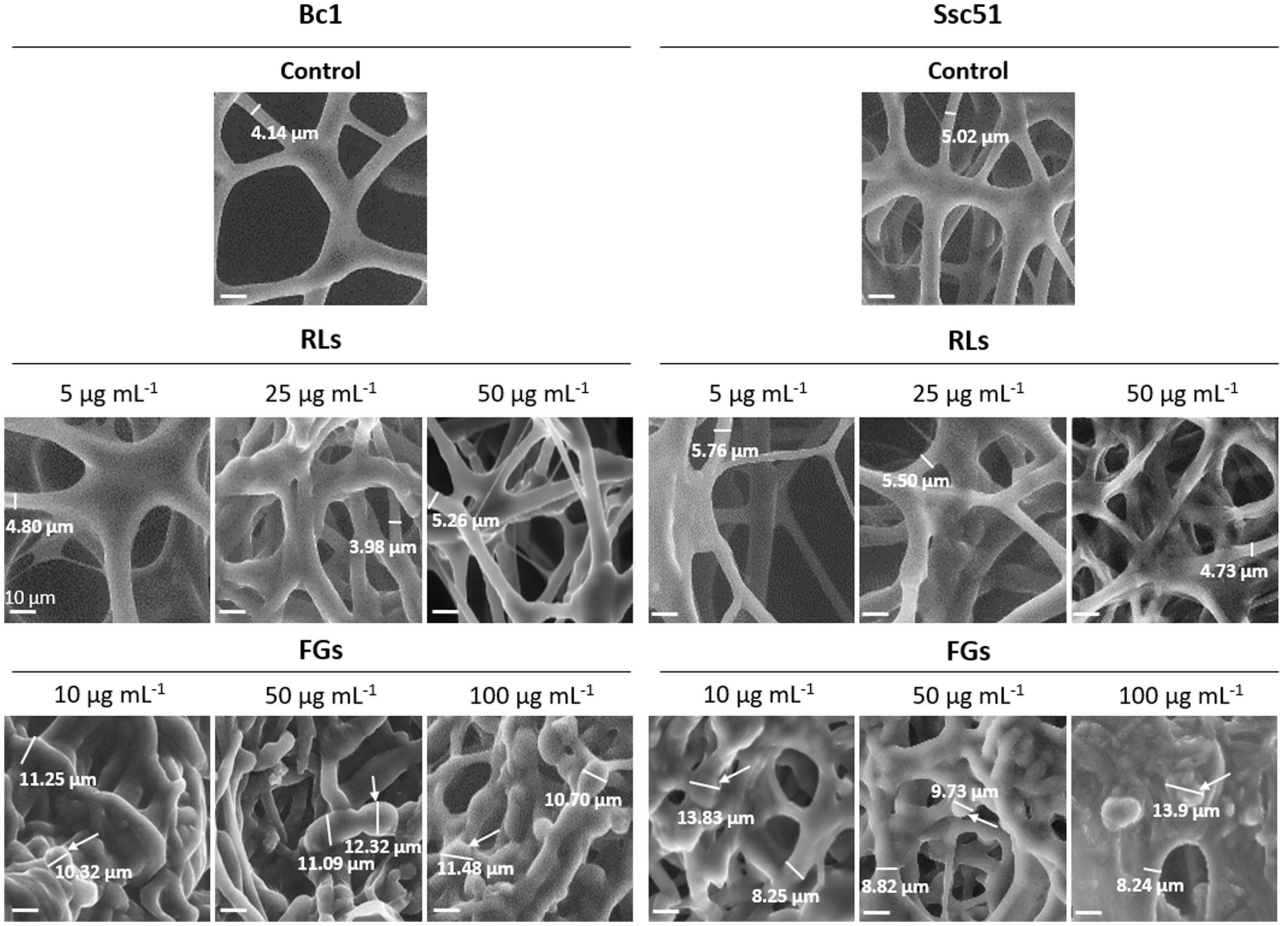
Structural alterations induced by RLs or FGs on *B. cinerea* Bc1 and *S. sclerotiorum* Ssc51 strains. Scanning electron microscopy observations of mycelium after treatments with different concentrations of RLs or FGs. The thickness of the mycelium is indicated by a measurement under a dash on each picture and vesicle-like structures are indicated by arrows. Scale bar: 10 μm.

To study the effects of RLs and FGs on the structure of hyphae, scanning electron microscopy observations on one strain of each fungal species grown in liquid growth medium were performed. Bc1 and Ssc51 were chosen as displaying representative contrasted sensitivity to both compounds in the mycelial growth experiments. Untreated mycelia exhibited cylindrical and regular shapes (Figure 2). The diameter of control hyphae was 4 to 5 μm. After 24 h of treatment with RLs, fusions and cluster formations of hyphae were observed on both fungi with increasing effects according to the concentration (5, 25 and 50 μg mL^−1^). The diameter of the hyphae was unchanged. FG-treated mycelium also displayed morphological changes at 10, 50 or 100 μg mL^−1^, with clear swelling and vesicle-like structure formations. The diameter of the hyphae increased by 1.5 to 2 times after FG adding and the vesicles measured 10 to 14 μm (Figure 2). No hyphal swelling or vesicle-like structures were observed after treatments with RLs even at 500 μg mL^−1^ (Supplementary Figure 1A). No differences in number of vesicle-like structures were observed between Ssc51 and Bc1 treated with 10 or 50 μg mL^−1^ of FGs. The number of structures also did not increase with the concentration on each fungal species (Supplementary Figure 1B).

### Induction of an autophagy mechanism by FGs but not by RLs

**Figure 3:**
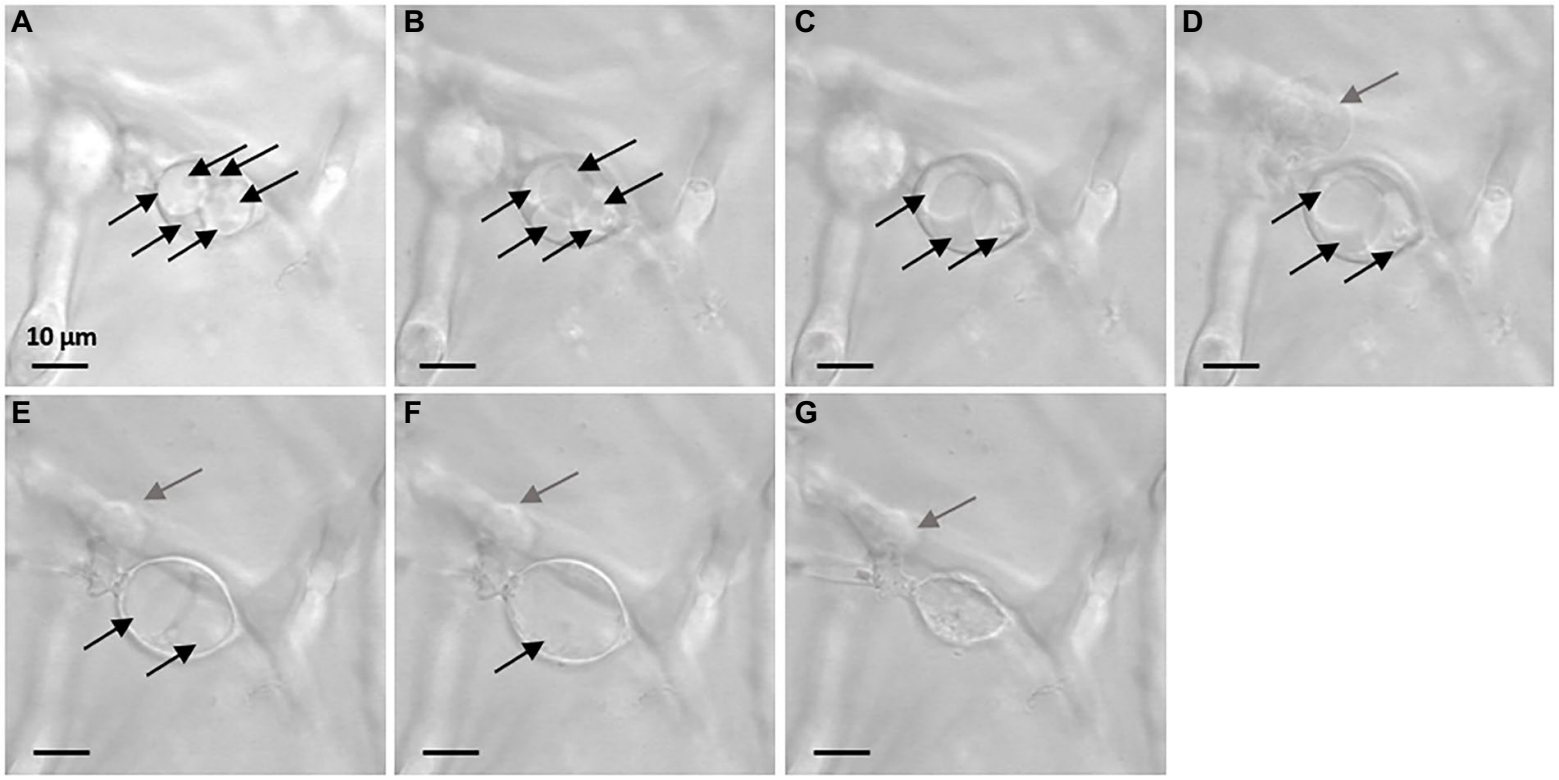
Evolution of vesicle-like structures induced by 50 μg mL^−1^ FGs on Ssc51 mycelium over time. The observation at *t* = 0 h (26 h after adding of FGs in the growth medium) shows 6 vacuoles inside the vesicle-like structure in the center of the picture **(A)**. At *t* = 1 h, there were 5 vacuoles **(B)**. At *t* = 1 h 49 min, there were 3 vacuoles in the structure **(C)**. At *t* = 1 h 57 min, a leakage from another vesicle-like structure was observed on the left of the picture **(D)**. At *t* = 4 h 20 min, 2 swollen vacuoles were observed in the central structure **(E)**. At *t* = 4 h 23 min, one vacuole remained **(F)**. At *t* = 4 h 43 min, the vesicle-like structure bursted and released its cytoplasmic content **(G)**. Each vacuole is indicated by a black arrow. Gray arrows indicate a leakage. Scale bar: 10 μm.

**Figure 4:**
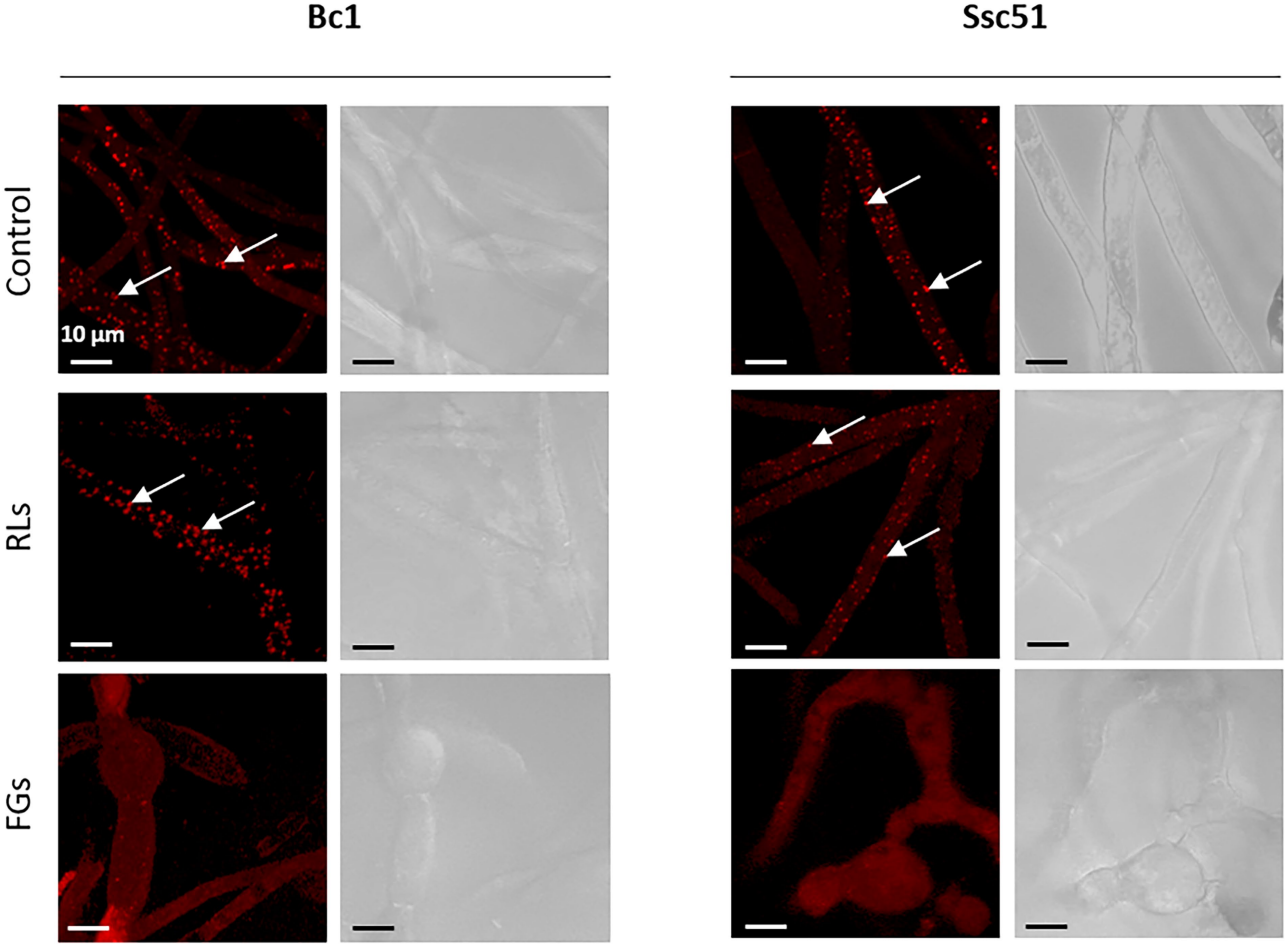
Nile red lipid droplets coloration of Bc1 and Ssc51 mycelium after 25 μg mL^−1^ RLs and 50 μg mL^−1^ FGs treatments. White arrows indicate lipid droplets. Scale bar: 10 μm.

Vesicle-like structures were observed by confocal microscopy on the Ssc51 strain treated with FGs at 50 μg mL^−1^ during a time lapse of 5 h. The vesicles contained several small vacuoles which swelled and merged over time leading to form one large vacuole (Figures 3A–F). These vesicles ended up bursting, resulting in total leakage of the cytoplasmic content (Figures 3D,G; Supplementary Video 1). Vacuole enlargement and fusions in filamentous fungi have generally been observed under autophagy triggered by many stresses (Zustiak et al., 2008). Autophagy is known to cause lipid droplet degradation (Zhang et al., 2018). A nile red staining of lipid droplets was then assessed on the mycelium of Ssc51 and Bc1 treated with 25 μg L^−1^ RLs or 50 μg L^−1^ FGs (Figure 4). In the control conditions, localized fluorescence was observed along hyphae indicating well defined lipid droplets for both fungi. A similar result was obtained after a RL treatment. With FGs, the fluorescence was more diffuse, showing the degradation of lipid droplets. This diffuse fluorescence was observed not only in vesicle-like structures but also almost everywhere in the rest of hyphae (Figure 4).

### Permeabilization and cell death induction of mycelial cells by RLs and FGs

**Figure 5:**
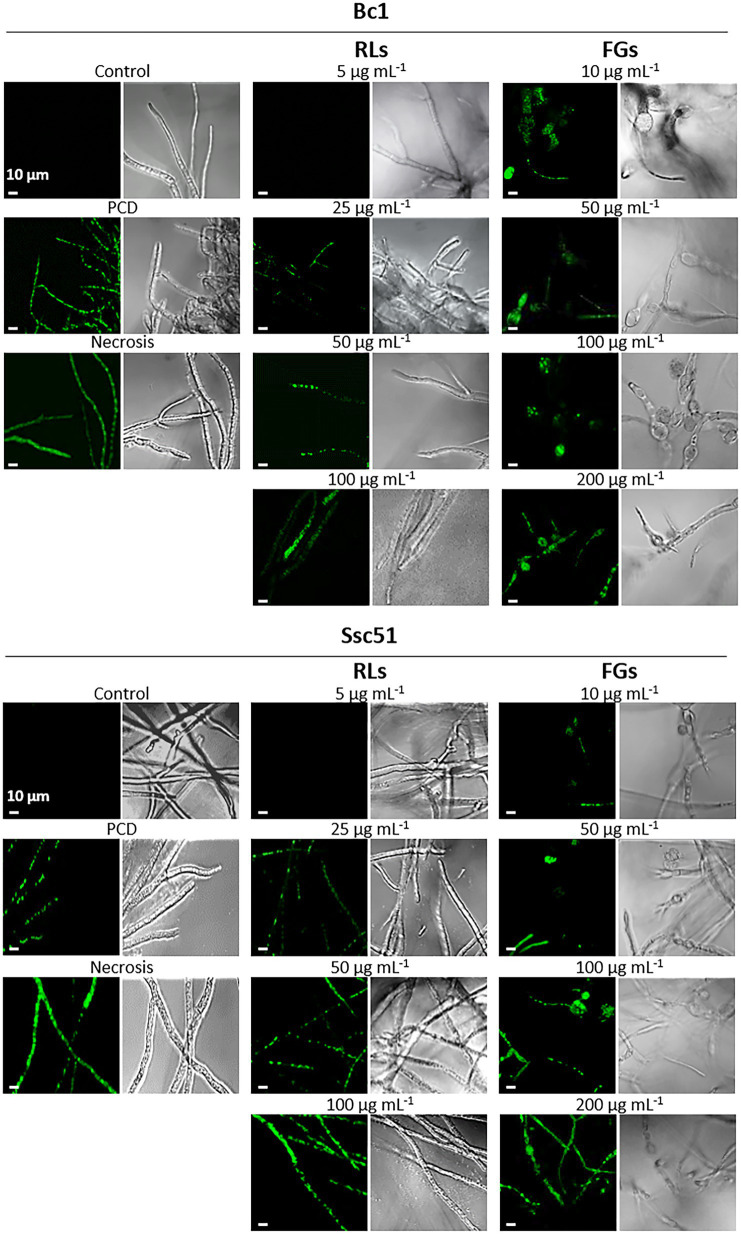
Permeabilization of mycelial cells by RLs or FGs. Confocal fluorescence microscopy observations of *B. cinerea* and *S. sclerotiorum* mycelium after treatments with different concentrations of RLs or FGs. PCD corresponds to mycelium treated with 3 mM hexanoic acid; necrosis corresponds to boiled mycelium. Cells were stained with SYTOX Green. Left panels are fluorescence microscopy images, right panels are light microscopy images. Scale bar: 10 μm.

To assess the integrity of biological membranes, cell permeabilization was studied by staining the mycelium with SYTOX green. This dye only penetrates permeable cells, inserts into nucleic acids and colors the nuclei in green (Thevissen et al., 1999). Observations by confocal microscopy showed that RLs, except at 5 μg mL^−1^, and FGs permeabilized the mycelium of *B. cinerea* and *S. sclerotiorum* whereas no permeability was observed in untreated controls (Figure 5). The fluorescence was localized in the nuclei, often fragmented, with medium concentrations (25 or 50 μg mL^−1^) of RLs and with all tested concentrations of FGs (10 to 200 μg mL^−1^). The staining was mainly located in the vesicle-like structures in this last case. A nucleus-localized coloration was also obtained in Bc1 and Ssc51 mycelium treated 3 h with 3 mM hexanoic acid, a compound known to induce the programmed cell death (PCD) mechanism (Finkelshtein et al., 2011). At a higher concentration of RLs (100 μg mL^−1^), the staining was more diffuse in hyphae, similar to what can be observed with mycelium boiled 10 min at 120°C and corresponding to a necrotic death. The staining of Bc1 appeared less intense than the staining of Ssc51 after a RL treatment. No differences in the fluorescence intensity were observed with FGs on both fungi (Figure 5).

**Figure 6:**
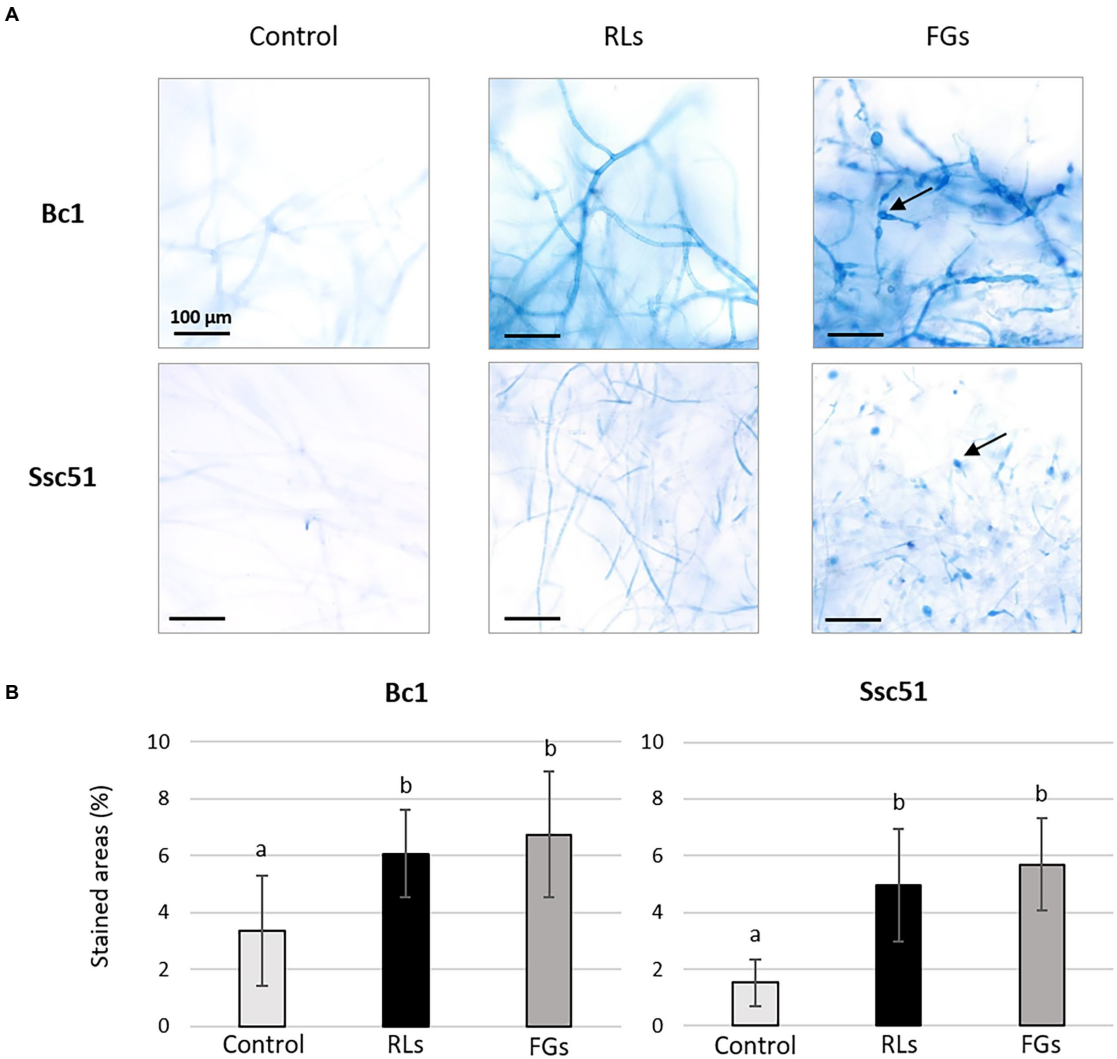
Evans blue dye coloration of Bc1 and Ssc51 mycelium after treatments with 25 μg mL^−1^ RLs or 50 μg mL^−1^ FGs. Light microscopy observations of stained mycelia, the arrows indicate vesicle-like structures. Scale bar: 100 μm **(A)**. Quantification of stained areas (%). Error bars represent standard errors. Letters indicate significant differences (*p* < 0.05) between the groups **(B)**.

A quantification with the Evans blue dye was also performed to compare the permeabilization induced by RLs and FGs. After staining imagery analysis, the results confirmed the occurrence of mycelium permeabilization by both compounds compared to the control conditions (Figure 6A) with no significant difference between a RL treatment at 25 μg mL^−1^ and a FG treatment at 50 μg mL^−1^ (Figure 6B). In the control conditions, few staining spots were observed, corresponding probably to natural senescence of the mycelia. Vesicle-like structures induced by FGs were stained in blue. Regardless of treatments, the stained areas were more localized in mycelia of Ssc51 than in those of Bc1, where stained areas were more diffuse (Figure 6A).

**Figure 7:**
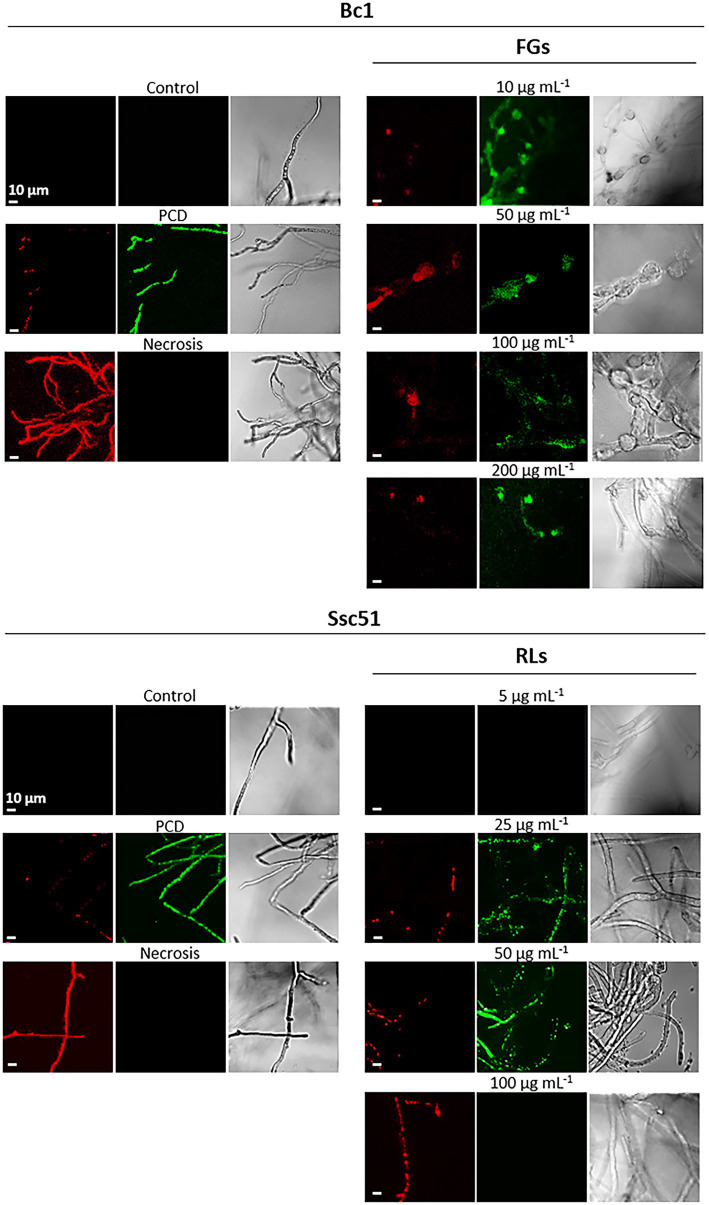
Cell death mechanisms after treatments with RLs or FGs. Confocal fluorescence microscopy observations of *B. cinerea* and *S. sclerotiorum* mycelium after treatments with different concentrations of RLs or FGs. PCD corresponds to mycelium treated with 3 mM hexanoic acid; necrosis corresponds to boiled mycelium. Cells were co-stained with propidium iodide (red) and Alexa Fluor^®^ 488 annexin V (green) to assess permeability and phosphatidylserine externalization, respectively. Left and middle panels are fluorescence microscopy images for propidium iodide and annexin V, right panels are light microscopy images. Scale bar: 10 μm.

We also assessed the death mechanisms induced by RLs and FGs using a dead cell apoptosis double coloration (Saibabu et al., 2017). The fluorochrome Alexa fluor® 488 combined with annexin V binds to phosphatidylserines externalized in the outer layer of the membrane during PCD and emits green fluorescence. The propidium iodide emits a red fluorescence when binding nucleic acids of permeable cells. It testifies to membrane permeability and highlights necrosis as observed on hexanoic acid-treated mycelium (Figure 7). It also highlights membrane permeabilization taking place at the end of PCD, when associated with a green emission of the Alexa Fluor® 488 fluorochrome (Hao et al., 2013). In our experiments, no red or green fluorescence was observed in untreated controls and in mycelia treated with RLs at 5 μg mL^−1^, indicating that the mycelial cells were alive. The green fluorescence observed by confocal microscopy confirmed PCD in Ssc51 treated with medium concentrations (25 and 50 μg mL^−1^) of RLs and a necrotic death at 100 μg mL^−1^ RLs, with all hyphae colored in red. At all tested concentrations of FGs, the Bc1 mycelium was colored in green, confirming the PCD traits (Figure 7). The same observation was made at 500 μg mL^−1^ of FGs (Supplementary Figure 2).

**Figure 8:**
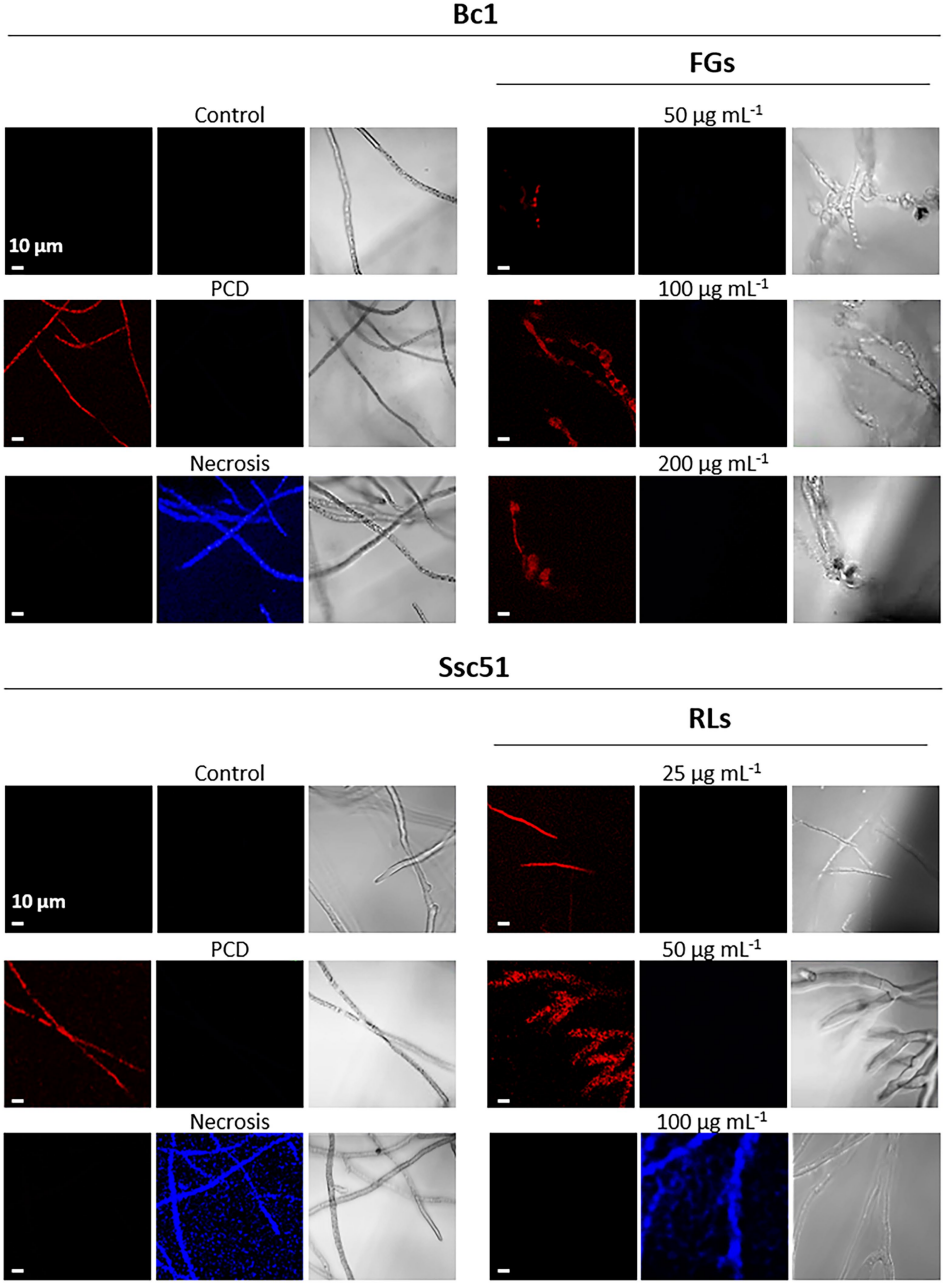
Induction of reactive oxygen species (ROS) production by RLs or FGs. Confocal fluorescence microscopy observation of Bc1 and Ssc51 mycelium after treatments with different concentrations of RLs and FGs. Cells were stained with dihydroethydium (DHE). PCD corresponds to mycelium treated with 3 mM hexanoic acid; necrosis corresponds to boiled mycelium. Left and middle panels are fluorescence microscopy images of oxidized DHE (red) and non-oxidized DHE (blue), right panels are light microscopy images. Scale bar: 10 μm.

The production of reactive oxygen species (ROS) is also induced during PCD establishment in fungi (Gonçalves et al., 2017). ROS production was measured by staining the mycelium with DHE. When the DHE is present in the cytoplasm, it emits a blue fluorescence. When ROS are produced, DHE is oxidized, it intercalates in the DNA and emits a red fluorescence. In our experiment, ROS production in treated mycelia was then evaluated by DHE oxidation and confocal microscopy observations. In this case, hexanoic acid-treated hyphae appeared red due to oxidized DHE accumulation and boiled mycelia appeared blue due to non-oxidized DHE accumulation (Figure 8). Untreated mycelium of both fungi exhibited neither red nor blue fluorescence, indicating that the mycelium did not produce ROS. The RLs clearly induced a production of ROS in *S. sclerotiorum* Ssc51 (red fluorescence), after 24 h of treatment at 25 and 50 μg mL^−1^. A treatment with 100 μg mL^−1^ RLs did not trigger ROS accumulation with non-oxidized DHE blue characteristics. On the contrary, FGs induced ROS production at all tested concentrations, confirming the induction of PCD (Figure 8).

### Role of ergosterol in the activity of RLs and FGs on *Botrytis cinerea* and *Sclerotinia sclerotiorum*

**Figure 9:**
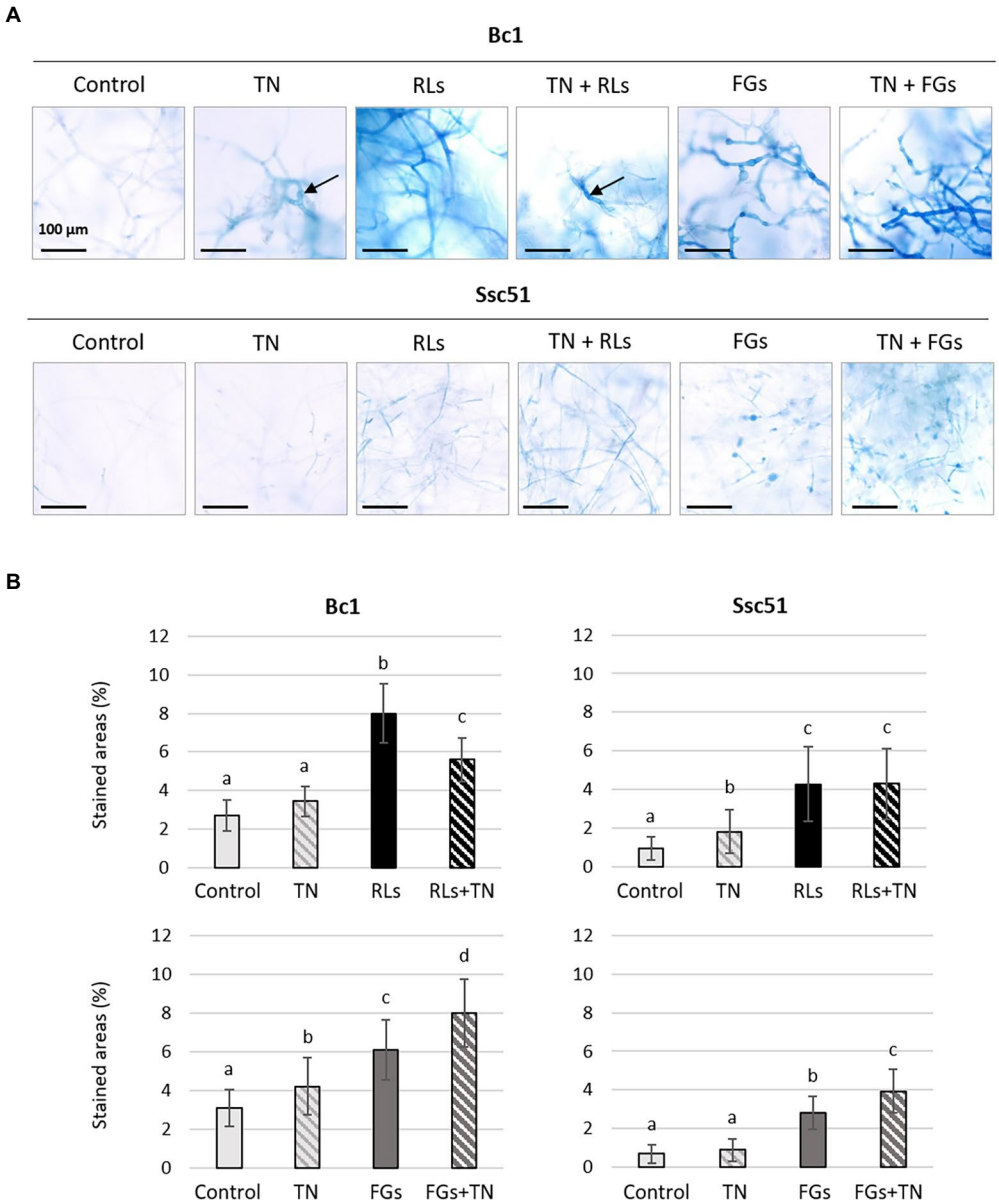
Evans blue dye coloration of Bc1 and Ssc51 mycelium after treatments with 25 μg mL^−1^ RLs or 50 μg mL^−1^ FGs alone or simultaneously with 1 mM TN. Light microscopy observations of stained mycelium. Scale bar: 100 μm. Black arrows show hyphal enlargement **(A)**. Quantification of stained areas (%). Error bars represent standard errors. Letters indicate significant differences (*p* < 0.05) between the groups **(B)**.

The plasma membrane lipid ergosterol has been proposed to play a role in the effects of FGs and RLs on plant pathogenic fungi (Mantil et al., 2019; Monnier et al., 2019). A treatment with 1 mM of the ergosterol biosynthesis inhibitor tebuconazole (TN; Price et al., 2015) was applied for 24 h on Ssc51 and Bc1 mycelium treated concomitantly with RLs or FGs in order to evaluate the ergosterol role in their activities. We then performed an Evans blue staining of the mycelium of both fungi to study the permeabilization. For Bc1, we observed hyphal enlargements in the TN-treated condition compared to the control condition. A decrease of the distance between two septa was also observed in these zones (Figure 9A). No morphological differences were observed between TN and control for Ssc51 hyphae. More stained areas were observed in TN-treated conditions than in control conditions but differences were not always significant for both fungi (Figures 9A,B). In the TN + RL treatment, no difference was observed on Ssc51 compared to the RL treatment. On the contrary, staining was less dark on Bc1 mycelium cotreated with TN + RLs than with a RL treatment alone. A darker staining was observed in TN + FGs than in FG conditions with a significantly higher percentage of stained areas for both fungi (Figures 9A,B). A SYTOX Green staining and confocal microscopy observations confirmed that the intensity of the fluorescence appeared higher when Bc1 was simultaneously treated with TN and FGs as compared to a treatment with FGs alone. On the contrary, no change was observed when Ssc51 was treated with RLs alone or TN and RLs simultaneously (Supplementary Figure 3A).

The TN sensitivity of the different strains of *B. cinerea* and *S. sclerotiorum* was also evaluated in mycelial growth experiments on PDA. At 1 mM, TN induced a radial growth inhibition of ca 50% for almost all fungal strains with few significant differences. The sensitivity of Bc26 to TN was lower with 37% of radial growth inhibition compared to the control (Supplementary Figure 3B).

**Figure 10:**
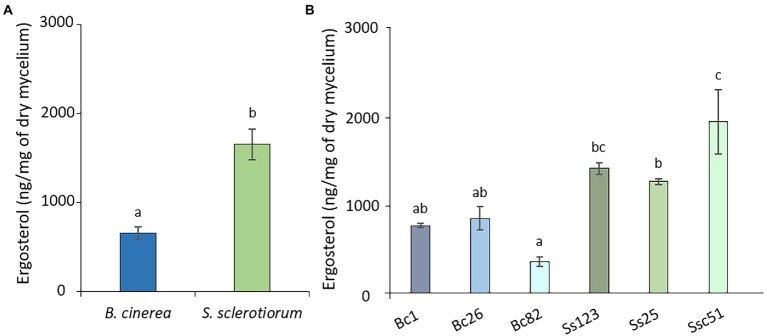
Ergosterol content of *B. cinerea* and *S. sclerotiorum* strains. Average ergosterol content of *B. cinerea* and *S. sclerotiorum* (means of 3 strains) **(A)**. Content of the different strains of *B. cinerea* (Bc1, Bc26, Bc82) and *S. sclerotiorum* (Ss123, Ss25, Ssc51) **(B)**. The height represents the mean value of at least 3 biological replicates. Error bars represent standard errors. Letters indicate significant differences (*p* < 0.05) between the groups.

We finally quantified the total ergosterol content in the mycelium of different strains of *S. sclerotiorum* and *B. cinerea*, using GC–MS. The mean ergosterol contents were 653.8 ± 68.1 ng mg^−1^ of dry weight and 1650.75 ± 172.63 ng mg^−1^ of dry weight, respectively for strains of *B. cinerea* and *S. sclerotiorum* (Figure 10A). There was no significant difference among the 3 strains of *B. cinerea*, but there was some discrepancy between the 3 strains of *S. sclerotiorum* with Ssc51 containing more ergosterol (Figure 10B).

## Discussion

In this work, the biocidal effects of RLs and FGs, two types of amphiphilic lipid compounds from bacterial secretomes, were studied on the plant pathogenic fungi *B. cinerea* and *S. sclerotiorum*. Despite their close taxonomic relatedness, these two species of the *Sclerotiniaceae* family display contrasted sensitivities to RLs and FGs, *B. cinerea* being roughly more sensitive to FGs and *S. sclerotiorum* being more sensitive to RLs. RLs have already been described for their capacity to inhibit the mycelium growth of *B. cinerea*. The results are difficult to compare due to different RL compositions, different *B. cinerea* strains and different growth conditions in different studies. Nevertheless, concentrations in the range of μg mL^−1^ are always described to inhibit the growth of the hyphae with a MIC (minimal inhibitory concentration) from 18 to 170 μg mL^−1^ (Kim et al., 2000; Abalos et al., 2001; Haba et al., 2003; Sha et al., 2012; Monnier et al., 2018). To give insights on the genotype effect of RL sensitivity, the growth of 3 strains of *B. cinerea* was compared here in the same conditions. Significant differences were obtained among the 3 strains. To our knowledge, the effect of RLs on *S. sclerotiorum* is described here for the first time and also show differences related to different fungal strains. FGs are reported as specifically active against filamentous fungi (Hu et al., 2007). They were previously described as inhibitor of *B. cinerea* mycelial growth with a MIC of 50 μg mL^−1^. The effect was lower on *S. sclerotiorum* mycelium with a MIC greater than 100 μg mL^−1^ (Vanittanakom et al., 1986). Our results, carried out under the same experimental conditions, showing that FGs are more active on *B. cinerea* than on *S. sclerotiorum*, are thus consistent with previous reports. It should be noted that, in our conditions, no difference of sensitivity to FGs has been observed between 3 strains of each fungus.

In the present work, morphological alterations of *B. cinerea* Bc1 and *S. sclerotiorum* Ssc51 hyphae structures were observed with both types of molecules. RLs led to fusions of hyphae, forming clusters and irregular shapes as described by Yan et al. (2015) on *Alternaria alternata* and Borah et al. (2016) on *Fusarium verticillioides*. The swelling of hyphae and vesicle-like structure appearing due to a FG treatment have already been described on *B. cinerea* and *S. sclerotiorum* (Vanittanakom et al., 1986; Alvarez et al., 2012; Farzand et al., 2019) and on other filamentous fungi such as *Ascodesmis sphaerospora, Monilinia fructicola, Paecilomyces variotii, Pyricularia oryzae, Rhizopus arrhizus, Stemphylium* sp.*, Tolypocladium inflatum, Mucor hiemalis* (Vanittanakom et al., 1986), *Colleotrichum acutatum* (Pathak and Keharia, 2014), *Magnaporthe grisea* (Zhang and Sun, 2018) and *V. inaequalis* (Desmyttere et al., 2019). Swelling and vesicle-like structure development thus appear as characteristic features of FG activity on different filamentous fungi species. Other lipopeptides, such as caspofungin on *Aspergillus fumigatus* (Bowman et al., 2002) or the bananamide group on *Magnaporthe oryzae* (Omoboye et al., 2019) are also known to induce this effect. These traits have never been observed with RLs even at a high concentration (500 μg mL^−1^) under our conditions or described in the literature.

Our work shows that the vesicle-like structure formation induced by FGs on *B. cinerea* and *S. sclerotiorum* goes with vacuole fusion and lipid droplets degradation. These effects are observed during the fungal autophagy mechanisms (Zustiak et al., 2008; Richards et al., 2010; Zhang et al., 2018). In eukaryotic cells, proteins or organelles that are damaged during the autophagic process are engulfed by autophagic vesicles with double membrane structures. They are shuttled to vacuoles in fungi for degradation and recycling (Zhu et al., 2019). Autophagy process occurs along the fungal life for development, nutrient starvation, pathogenicity (Zhu et al., 2019) and antibiotic action (Kim et al., 2011). In yeast, vacuoles play a role in lipid droplets degradation under autophagy by internalizing them, leading to a lipophagy mechanism by lipases (Van Zutphen et al., 2014) to produce the needed cell components (Xie et al., 2020). Then, autophagy can serve to repair the cells. If autophagy fails to the cell repairing, autophagic or apoptosis death can occur (Lockshin and Zakeri, 2004). Thus, the autophagy process induced by FGs appears here as a cell defense mechanism in order to respond to a toxicity. Interestingly, the top of *S. sclerotiorum* hyphae grown on PDA plates containing the fermentation broth of *Bacillus amyloliquefaciens*, a bacteria producing lipopeptides, have been described as abnormal, swollen, or turned into “balloons.” The transcriptomic study performed on *S. sclerotiorum* under *B. amyloliquefaciens* stress has also shown that autophagy-related genes and a gene related to a vacuolar lipase were upregulated (Yang et al., 2020). Nevertheless, this autophagy mechanism associated to vacuole enlargement and vesicle-like structure formation does not seem specific to membrane targeting lipopeptides since olorofim, selectively inhibiting the *de novo* pyrimidine biosynthesis of filamentous fungi, is described to trigger similar effects on *A. fumigatus* (Du Pré et al., 2020).

*S. sclerotiorum* strains are less sensitive to FGs than *B. cinerea* strains in plate experiments, regardless of the vesicle-like structure induction in both fungi in liquid growth medium. Indeed, no significant differences between the number of vesicle-like structures induced by FGs on *S. sclerotiorum* or *B. cinerea* were observed. Then, the development of these structures does not seem to be fully correlated to the antifungal effect of the compounds. Similarly, the number of structures is not influenced by the concentration of FGs either, since *S. sclerotiorum* or *B. cinerea* treated with 10 or 50 μg mL^−1^ developed the same number of structures.

Vesicle-like structures are not systematically reported on filamentous fungi treated with bacterial lipopeptides. Indeed no round shape observations were reported after a treatment with the iturin bacillomycin L on *Rhizoctonia solani* (Zhang et al., 2013), nor fengycin, iturin A, bacillomycin and surfactin on *Podosphaera fusca* (Romero et al., 2007) or mycosubtilin on *Fusarium graminearum* (Yu et al., 2021). On the contrary, mycosubtilin induced subterminal vesicles and intercalary swelling on *F. oxysporum* mycelium (Mihalache et al., 2018). Yan et al. (2020) reported that in the presence of iturin A, most of the *Colletotrichum gloeosporioides* mycelium expanded into a sphere. After treatment with iturin A, a few balloon-shaped cells were observed in *F. graminearum* (Kim et al., 2017). Iturin and mycosubtilin can act as antifungals with or without the induction of spherical structures in the mycelium (Romero et al., 2007; Zhang et al., 2013; Kim et al., 2017; Yan et al., 2020). The produced structures can differ in shapes (Desmyttere et al., 2019). Other parameters seem to influence the mechanisms as Yu et al. (2021) did not report balloon-shaped cells in *F. graminearum* after a treatment with Iturin A whereas Kim et al. (2017) reported them. Then, these kinds of structures seem to differ in nature and to depend on the fungus, rather than on a general antifungal mode of action of lipopeptides.

SYTOX green and Evans blue staining showed that RLs and FGs permeabilize and kill the fungal cells of *B. cinerea* Bc1 and *S. sclerotiorum* Ssc51. Nevertheless, the SYTOX green nucleic acid staining reveals different effects depending on the concentration of RLs. A diffuse stain, obtained with RLs in the concentration of about 100 μg mL^−1^, suggests DNA degradation and necrosis as observed in mycelium after boiling. A stain localised to well defined nuclei is observed with 25 or 50 μg mL^−1^ and with an hexanoic acid treatment inducing PCD. These results were confirmed with observations of phosphatidylserines externalisation in the outer leaflet of the fungal membranes and ROS production confirming PCD induced by 25 or 50 μg mL^−1^ of RLs. In our experiments, FGs induced PCD whatever the concentration. Interestingly, Tang et al. (2014) described the necrotic effect of FGs on *Schizophyllium commune* at concentrations higher than 50 μg mL^−1^. This may indicate different death processes triggered by FGs depending on the species or environmental conditions.

Ergosterol is the major sterol found in fungal membrane (Wassef, 1977; Weete et al., 1985) and plays a role in the dynamic, the rigidity, the fluidity and the permeability of the membrane (Rattray et al., 1975; Ermakova and Zuev, 2017). Plasma membrane fluidity can determine fungal sensitivity to membranotropic compounds and is involved in the permeabilization of the cells (Palma-Guerrero et al., 2010). Loss of membrane integrity can lead to changes in the cells such as permeability, cell death mechanism inductions, modification of the mycelium structure and inhibition of the fungal growth (Thevissen et al., 1996; Mello et al., 2011; Yan et al., 2015; Taveira et al., 2017). Ergosterol has been proposed to play a role in fungal sensitivity towards FGs. Indeed, a low ergosterol content in fungi could decrease the capacity to buffer the fluidity induced by FGs (Wise et al., 2014). Moreover, biophysical studies on fungal membrane models containing ergosterol show increased membrane leakages in presence of FGs associated with low or no ergosterol (Mantil et al., 2019). In our study, *B. cinerea* has a lower ergosterol content and is more sensitive to FGs than *S. sclerotiorum*, which corroborates the hypothesis from previous work. On the contrary, it has been shown on fungal membrane models that a higher membrane fluidity is observed in the presence of RLs (Monnier et al., 2019). The total ergosterol content of *S. sclerotiorum* is about twice as high as that of *B. cinerea*, which is also the most sensitive fungus to RLs on plate experiments. Both hypotheses were confirmed by the TN assays showing an increase of permeability on Bc1 and Ssc51 hyphae treated with FGs and TN simultaneously, as well as the decrease or the absence of effect of permeability induced by a RLs and TN cotreatment on Bc1 and Ssc51, respectively. Our results therefore confirm the opposite effect of the mycelium ergosterol content on RL and FG activities *in vivo*.

Considering the different strains, Bc26 appears less sensitive to RLs than Bc82 which contains less ergosterol. Additionally, Ssc51 contains more ergosterol than Ss25 which is less sensitive to RLs. This could indicate the implication of other membrane parameters to explain antifungal activity of RLs. For instance, the fatty acid lengths or the anionic/zwitterionic phospholipid ratio are proposed to play a role in sensitivity to FGs (Wise et al., 2014). Global lipid analysis will be necessary to fully understand the implication of the lipid part of the fungal plasma membrane in the interactions with RLs and FGs.

In our results, treated mycelium with TN show hyphal enlargement in Bc1 but not in Ssc51 strain. Kang et al. (2001), also described swollen hyphae after TN treatment on *Fusarium culmorum* mycelium as well as septa very close together compared to the hyphae from untreated control. Our results also show a lesser quantity of ergosterol in Bc1 than in Ssc51. Knowing that TN inhibits the ergosterol synthesis, there could be a link between ergosterol content and morphological changes. Moreover, triazole resistance has been observed with a reduction of ergosterol content in the mycelium of *V. inaequalis* treated with penconazole (Vijaya Palani and Lalithakumari, 1999). Bc26 strain is the most resistant to TN in our case but it has an ergosterol rate similar to Bc1 and Bc82 strains. The *S. sclerotiorum* strains are similarly more sensitive to TN than Bc1 and Bc82 but they contain more ergosterol. Then, our results do not indicate a correlation between TN sensitivity on plate experiments and ergosterol contents of *B. cinerea* and *S. sclerotiorum*.

Finally, the activity of FGs was previously described as very similar to the activity of TN (Desmyttere et al., 2019). Yet, our results do not show a correlation between the antifungal activities of FGs and TN on *S. sclerotiorum* and *B. cinerea*. On the contrary, it is noticeable that RLs and TN give similar results on plate experiments, Bc26 being more resistant than other strains to both compounds. If we cannot exclude an indirect effect of RLs on the ergosterol fungal content, this would remain to be studied. However, other factors could influence the resistance of fungi to TN. For example, an important presence of membrane transporter proteins involved in drug resistance by an efflux mechanism could reduce the sensitivity (Cools et al., 2013). Most of all, our work clearly highlights the differences in the mode of action of TN and RLs or FGs with no permeabilization observed with TN in Evans blue or SYTOX Green colorations confirming the interest of the membranotropic lipid-based antifungal compounds regarding to azole compounds.

Altogether our results indicate a different mode of action of the amphiphilic lipid-based compounds RLs and FGs on filamentous fungi which have both been proposed to act through a direct interaction with the plasma membrane of microorganisms. The implication of ergosterol content in the antifungal activity here is confirmed for both metabolites. The fact that RLs and FGs have different effect on the closely related fungi *S. sclerotiorum* and *B. cinerea* must be considered to wisely apply them to fight specific plant diseases as direct antifungal compounds.

**Figure 11:**
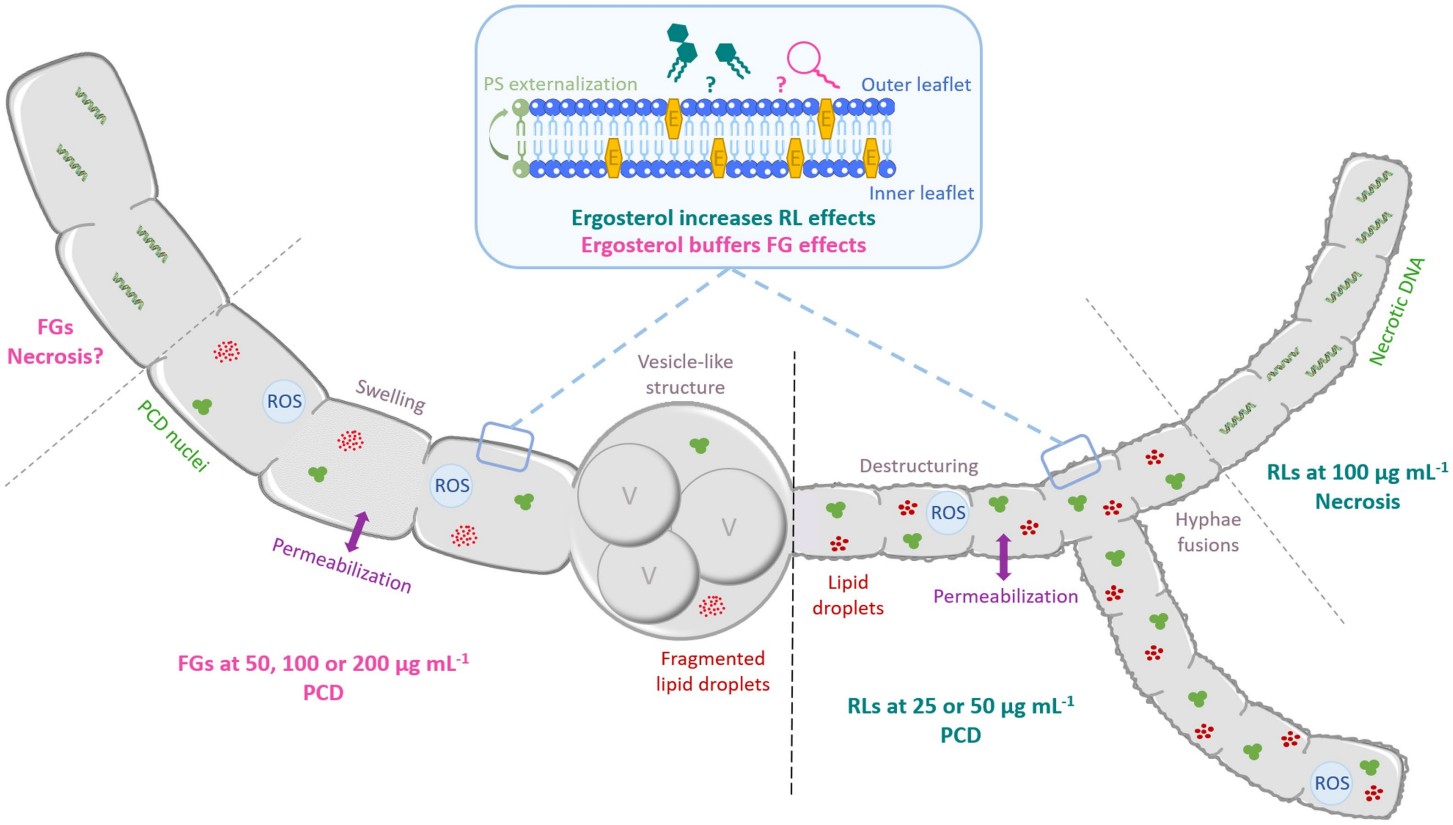
Schematic representation of the antifungal mode of action of RLs and FGs on *B. cinerea* and *S. sclerotiorum* hyphae according to the concentration. Both compounds induce hyphae permeabilization. RLs induce destructuring and fusion of hyphae. FGs trigger swelling and formation of vesicle-like structures associated to vacuole fusion and lipid droplets degradation. At medium concentration (until 50 μg mL^−1^), RLs induce PCD with condensed DNA and fragmented nuclei, ROS production and PS externalization. At 100 μg mL^−1^, RLs induce necrotic death with DNA fragmentation. FGs trigger PCD at all concentration tested. E: ergosterol, PCD: programmed cell death, PS: phosphatidylserine, ROS: reactive oxygen species, V: vacuole.

A scheme illustration to summarize the antimycelial mode of action of RLs and FGs on *S. sclerotiorum* and *B. cinerea* is proposed Figure 11.

## Data availability statement

The raw data supporting the conclusions of this article will be made available by the authors, without undue reservation.

## Author contributions

CB, TB, CS, and SR: conceived and designed the experiments. CB and TB: performed the experiments and different measurements. SA: initiated the GC–MS analyses with CB. DL, CD, AR, and VL: have been in charge of the production, purification and analyses of the FGs. PN: provided some of the different strains of fungi from the UPV bank and validated the growth culture conditions. CB, TB, CS, and SR: analysed the data and wrote the manuscript. All authors helped with drafting the manuscript and approved the final version.

## Funding

This research was supported by the Hauts-de-France Council and was carried out in the context of the COALA project. Camille Botcazon PhD thesis was co-funded by the HdF Council and the French Ministry of Higher Education, Research and Innovation (MESRI). Thomas Bergia phD was funded by the MESRI. This study was supported by equipments co-founded by the Regional Council of Picardy and European Union within the CPER 2007–2020.

## Conflict of interest

The authors declare that the research was conducted in the absence of any commercial or financial relationships that could be construed as a potential conflict of interest.

## Publisher’s note

All claims expressed in this article are solely those of the authors and do not necessarily represent those of their affiliated organizations, or those of the publisher, the editors and the reviewers. Any product that may be evaluated in this article, or claim that may be made by its manufacturer, is not guaranteed or endorsed by the publisher.
