# Supplementary figures and images for: Rhamnolipids and fengycins, very promising amphiphilic antifungal compounds from bacteria secretomes, act on Sclerotiniaceae fungi through different mechanisms
Source: Front Microbiol. 2022 Sep 29;13:977633. doi: 10.3389/fmicb.2022.977633 (PMC9557291; doi:10.3389/fmicb.2022.977633)

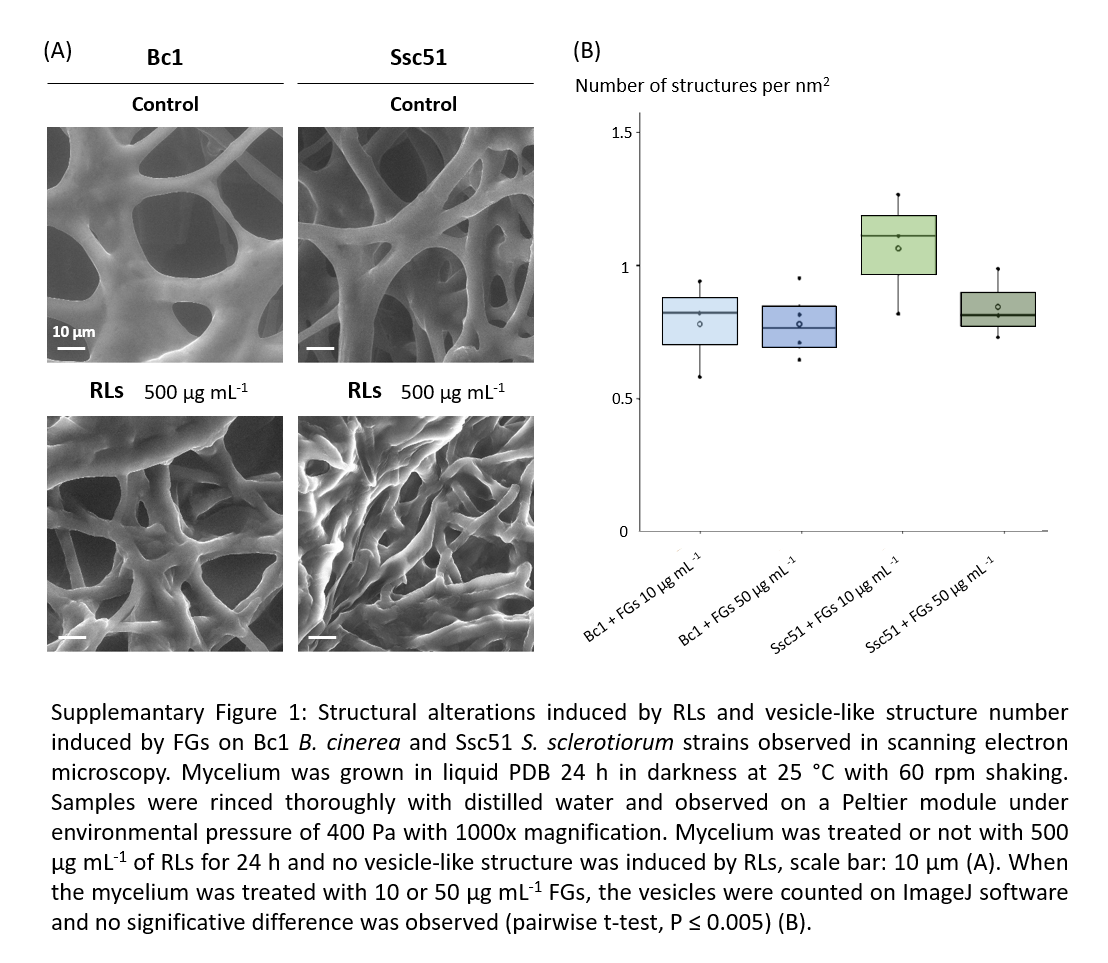

Supplement: Supplementary file 1 [file Image_1.TIF]

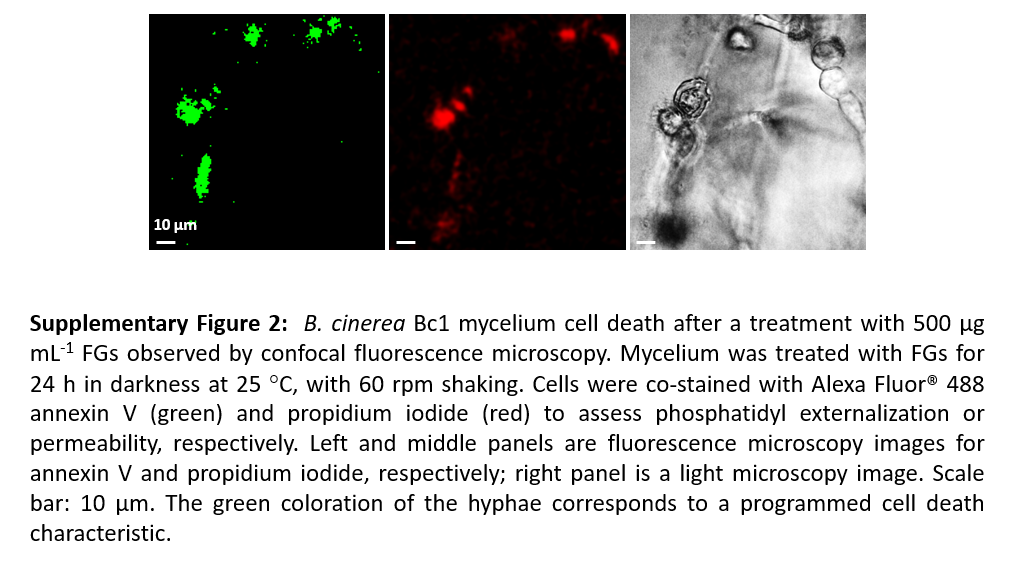

Supplement: Supplementary file 2 [file Image_2.TIF]

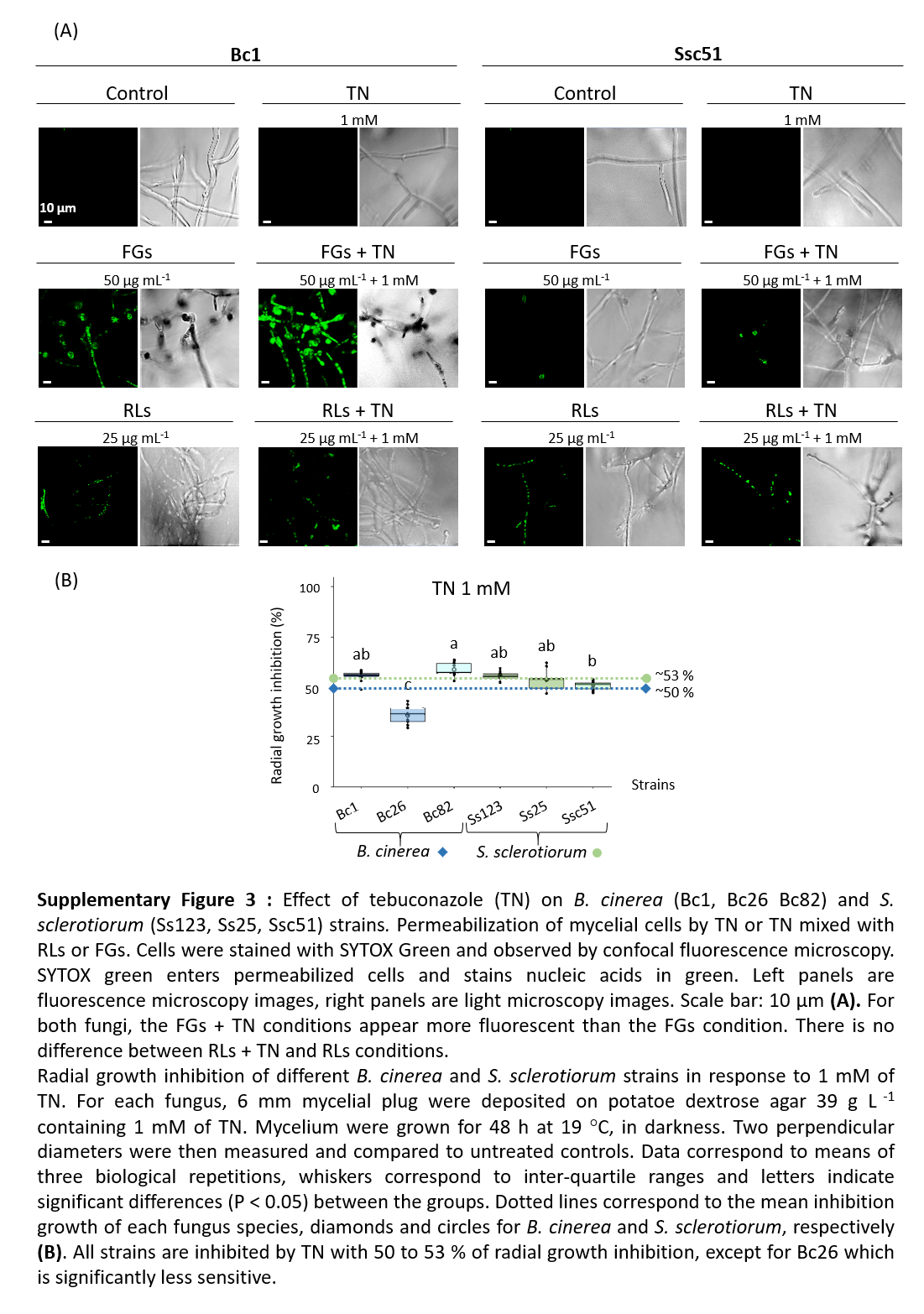

Supplement: Supplementary file 3 [file Image_3.TIF]
